# miR-124-3p Combined with ANGPTL2 Has High Diagnostic Values for Obese and Nonobese Polycystic Ovary Syndrome

**DOI:** 10.1155/2022/2155018

**Published:** 2022-06-14

**Authors:** Hongmei Dai, Fangting Liu, Jianshu Lu, Yan Yang, Pingping Liu

**Affiliations:** ^1^Department of Reproductive Medicine, Dongying People's Hospital, Dongying, Shandong, China; ^2^Department of Obstetrics and Gynecology, Dongying People's Hospital, Dongying, Shandong, China; ^3^Department of Orthopaedics, Dongying People's Hospital, Dongying, Shandong, China; ^4^Department of Respiratory, Dongying People's Hospital, Dongying, Shandong, China

## Abstract

Polycystic ovary syndrome (PCOS) is a hormonal disorder that affects 5–20% of women of reproductive age. Interestingly, serum miR-124-3p and ANGPTL2 are differentially expressed in PCOS patients. Accordingly, this study set out to explore the clinical roles of serum miR-124-3p/ANGPTL2 in PCOS. Firstly, miR-124-3p/ANGPTL2 expression patterns were detected in the serum of 102 PCOS patients and 100 healthy subjects. miR-124-3p or/and ANGPTL2 diagnostic efficacy on PCOS was further analyzed, in addition to the measurement of lipid metabolism, glucose metabolism, sex hormone indexes, and inflammation levels. Correlations between serum miR-124-3p/ANGPTL2 expressions and age, BMI, Ferriman–Gallwey score, lipid metabolism, glucose metabolism, sex hormone indexes, TNF-*α*, and IL-6 in PCOS patients were determined. The expression correlation and binding relationship of ANGPTL2 and miR-124-3p were identified. In addition, miR-124-3p was downregulated and ANGPTL2 was upregulated in the serum of obese and nonobese PCOS patients. miR-124-3p expression was found to be negatively correlated with Ferriman–Gallwey score and serum total testosterone (T), and negatively related to prolactin (PRL). ANGPTL2 expression was positively correlated with FNS and inversely linked with PRL. TNF-*α* and IL-6 were negatively correlated with miR-124-3p, but positively correlated with ANGPTL2. Furthermore, there was a negative correlation and a targeting relationship between ANGPTL2 and miR-124-3p expression in the serum of obese and nonobese PCOS patients. Collectively, our findings indicated that miR-124-3p might target ANGPTL2 expression in obese and nonobese PCOS patients, and further underscored the diagnostic value of their combination.

## 1. Introduction

Obesity is a chronic disease strongly associated with a low but prolonged inflammatory state characterized by the presence of increased cytokines in blood and tissues, such as tumor necrosis factor-*α* (TNF-*α*) and interleukin-6 (IL-6), all of which precipitate insulin resistance in some tissues and ultimately cause type 2 diabetes mellitus and other health complications [1]. Interestingly, there is compelling evidence illustrating that polycystic ovary syndrome (PCOS) patients affected by obesity exhibit high levels of TNF-*α* and IL-6 in serum and adipose tissues [2, 3]. PCOS is the most prevalent endocrine disorder and is characterized by anovulation, polycystic ovaries, and hirsutism [4]. According to current international guidelines, the “Rotterdam” diagnostic criterion requires at least two of the following features for the clinical diagnosis of PCOS: hyperandrogenism, ovulatory dysfunction, or/and polycystic ovaries during ultrasonography, and exclusion of other pathologies [5]. PCOS is a multifactorial disease related to genetic and environmental factors and associated with several factors, such as obesity, insulin resistance, glucose tolerance, dyslipidemia, and diabetes [6–8]. Moreover, insulin resistance is known to play a critical role in the development and persistence of PCOS [9]. Similarly, PCOS is strongly associated with obesity, with 38%–88% of PCOS patients being either obese or overweight [10]. Unsurprisingly, our peers have hypothesized that there is bidirectional interaction between obesity and PCOS [11]. Obesity in turn aggravates PCOS symptoms, and body mass index (BMI) shows the strongest association with PCOS status, with a 9% increased risk of PCOS for each additional BMI point [12]. Other long-term consequences of PCOS also include diabetes, cardiovascular disease, and hyperlipidemia [6]. Moreover, PCOS is regarded as a chronic inflammatory state accompanied by elevated white blood cells, pro-inflammatory cytokines, and white blood cell counts, which also affects women with normal BMI [13–15]. In addition, numerous authors have stressed the role of inflammation in PCOS, such that elevated levels of inflammatory markers such as TNF-*α*, IL-6, and IL-18, are directly related to the occurrence and development of PCOS [16]. Additionally, an increase in serum IL-8 levels in PCOS patients is positively correlated with BMI, insulin resistance index, and serum testosterone (T) levels [17]. However, the underlying pathophysiology of PCOS remains elusive.

Angiopoietin-like protein 2 (ANGPTL2), a kind of 64-kDa glycosylated protein that belongs to the angiopoietin-like family, is known to be capable of promoting angiogenesis and anti-apoptosis as it contains a fibrinogen-like domain similar to angiopoietin in structure [18]. In addition, ANGPTL2 is established as a pro-inflammatory protein associated with states of chronic inflammation, such as diabetes, atherosclerosis, and cancer, and is further widely expressed in visceral adipose tissues [19]. What's noteworthy is that ANGPTL2 also exerts pivotal roles in angiogenesis, androgen biosynthesis, adipocyte function, and insulin resistance [20]. Various clinical studies have revealed that the ANGPTL2 level significantly increases in a variety of chronic inflammatory diseases and is related to the diagnosis and prognosis of chronic kidney disease, diabetes, cardiovascular diseases, and a variety of cancers [21]. Existing evidence further indicates that ANGPTL2 is an essential inflammatory mediator derived from adipocytes, which links systemic insulin resistance to obesity and induces inflammation and insulin resistance [22]. Moreover, previous studies have documented the participation of ANGPTL2 in PCOS development via the PI3K/Akt pathway [23]. The aberrant expression of ANGPTL2 in cumulus cells is further related to the impaired developmental ability of oocytes in PCOS, which underscores the significance of ANGPTL2 in PCOS [20].

microRNAs (miRNAs), a type of small noncoding single-stranded RNA molecules of 18–24 nucleotides in length, are well known for their ability to directly regulate gene expression after transcription, which target the 3'untranslated region of mRNAs, resulting in the suppression of mRNA expression and ultimately contributing to the inhibition of protein translation to regulate cellular physiological functions [24]. What's more, miRNA expression profiles are altered in the ovary in the states of PCOS [25]. Furthermore, preceding research has unveiled three kinds of miRNAs capable of serving as PCOS biomarkers [26], and 29 miRNAs that are aberrantly expressed in normal and PCOS women [24]. One such miRNA, namely, miR-124-3p, is significantly downregulated in PCOS patients and further indicated as a potential therapeutic target for PCOS [27]. Moreover, miR-124-3p regulates NF-*κ*B, TNF-*α*, IL-6, and metalloproteinase-7 (MMP-7) to inhibit inflammatory responses [16]. At present, limited studies have explored the expressions of ANGPTL2 and miR-124-3p in the serum of patients with PCOS and their clinical significance. In addition, the possible association of serum ANGPTL2 and miR-124-3p with obesity, inflammation, insulin resistance, and endocrine hormone levels in PCOS patients, and their potential to serve as biomarkers for the diagnosis of PCOS remain to be further studied. In lieu of the same, the current study sought to investigate the expressions and clinical significance of ANGPTL2 and miR-124-3p in the serum of obese and nonobese PCOS subjects, in an effort to provide certain reference values for the diagnosis and treatment of PCOS.

## 2. Materials and Methods

### 2.1. Ethics Statement

The current study was authorized by the academic ethics committee of Dongying People's Hospital. All experimental procedures were carried out in strict adherence to the code of ethics. Signed informed consents were obtained from all participants prior to sampling.

### 2.2. Study Subjects

A total of 102 patients who were diagnosed with PCOS at the outpatient department of obstetrics and gynecology of Dongying People's Hospital from June 2018 to July 2021 were recruited as the PCOS group. All included PCOS patients met the relevant standards from ESHRE/ASRM Rotterdam meeting [28]. In accordance with the Asia-Pacific criteria (BMI ≥25 indicates obesity) proposed by WHO 2000 International Obesity Task Force [29], the enrolled PCOS patients were assigned to the PCOS-obese group (*N* = 42) and PCOS-nonobese group (*N* = 60). Additionally, 100 age-matched healthy women who underwent health examination at Dongying People's Hospital during the same period were enrolled to serve as the control group and allocated into the control-obese group (*N* = 32) and the control-nonobese group (*N* = 68). The control group subjects presented regular menstrual cycle functioning and normal ovarian morphology. All PCOS patients and the control subjects presented with no tumors, no cardiovascular disease, or no autoimmune disease, no recent infection history, and no history of taking oral contraceptives, hypolipidemic drugs, hypoglycemic drugs, and glucocorticoids in the past 3 months. Baseline data of the enrolled subjects including age, systolic blood pressure, diastolic blood pressure, BMI, and hirsutism were collected. Hirsutism was evaluated using the Ferriman–Gallwey scoring system as follows: grade 1 was 1 score, grade 2 was 2 score, and so on, with the total score <6 indicating normal; 6–9 indicating excessive hair; and >9 indicating hirsute.

### 2.3. Serum Sample Collection

Fasting peripheral venous blood samples were collected from all subjects after fasting for 8 h on the 5th to 8th day of the menstrual cycle (the time for patients with amenorrhea was unlimited) and sitting for 30 min. The collected samples were centrifuged at 3000 r/min for 15 min to obtain the serum, which was stored in a refrigerator at 4°C. Serum hormones and biochemical indexes were subsequently detected on the same day, and the remaining serum was packed and stored at −20°C for further experimentation.

### 2.4. Biochemical Index Detection

A Hitachi 7100 biochemical analyzer was adopted to analyze serum triglyceride (TG), total cholesterol (TC), and other lipid metabolism indexes. Fasting blood glucose (FBG) was measured using the glucose oxidase method. Prolactin (PRL), estradiol (E2), follicle-stimulating hormone (FSH), T, luteinizing hormone (LH), and fasting insulin (FINS) levels were detected with the Roche Cobas-e-602 electrochemiluminescence immunoassay system. Insulin resistance index HOMA-IR = FINS (mU/L) × FBG (mmol/L)/22.5. Additionally, serum ANGPTL2 (Human ANGPTL2 ELISA kit, SND-H1948, Shinuoda Biological Technology, Chuzhou, Anhui, China), IL-6 (Human IL-6 ELISA Kit, D711013-0048, BBI, Shanghai, China), and TNF-*α* (HUMAN TNF ALPHA ELISA (2-PAK), EH3TNFA2, Invitrogen, Carlsbad, CA, USA)levels were detected by an enzyme-linked immunosorbent assay (ELISA) in strict accordance with the provided instructions.

Total RNA extraction and reverse-quantitative polymerase chain reaction (RT-qPCR): the TRIzol reagent (Invitrogen) was adopted to extract the total RNA content. The obtained RNA was reverse-transcribed into cDNA using PrimeScript RT reagent kits (Takara, Dalian, China). The qPCR was subsequently performed on the ABI7900HT fast PCR real-time system (Applied Biosystems, Foster city, CA, USA) using SYBR® Premix Ex Taq™ II (Takara). The reaction conditions were as follows: pre-denaturation at 95°C for 5 min and 40 cycles of denaturation at 95°C for 15 s, annealing at 60°C for 20 s, and extending at 72°C for 35 s. GAPDH and U6 were utilized as the internal parameter, and the 2^−ΔΔCt^ method was adopted for gene expression analyses. The primer sequences (synthetized by Sangon Biotech Co., Ltd., Shanghai, China) are illustrated in Table 1.

### 2.5. Dual-Luciferase Reporter Assay

The binding sites of miR-124-3p and ANGPTL2 were predicted using the StarBase database (https://starbase.sysu.edu.cn/). Subsequently, the complementary binding sequences and mutated sequences of miR-124-3p and ANGPTL2 were amplified and cloned into the pmiRGLO dual-luciferase reporter gene vector (Promega, Madison, WI, USA) to construct wild-type plasmid pmiRGLO-ANGPTL2-WT and the corresponding mutant plasmid pmiRGLO-ANGPTL2-MUT. In accordance with the instructions of Lipofectamine^TM^ 2000 (Invitrogen), the plasmid was cotransfected with mimic NC or miR-124-3p mimic (sequence: 5′-UAAGGCACGCGGUGAAUGCCCA-3′, purchased from GenePharma, Shanghai, China) into ovarian granulosa cells KGN (Sunncell, Wuhan, Hubei, China). Afterwards, the luciferase activity was detected 48 h later.

### 2.6. Statistical Analysis

SPSS 21.0 (IBM Corp. Armonk, NY, USA), MedCalc (MedCalc® statistical software), and GraphPad Prism 8.01 (GraphPad Software Inc) software were adopted for data analyses and mapping. Normal distribution of continuous variables was verified with the Shapiro–Wilk test. Variable data were expressed as mean ± standard deviation, and the categorical variables were expressed in counts and percentages. The independent *t*-test was utilized for comparisons between two groups. One-way analysis of variance (ANOVA) was adopted for comparisons among multiple groups and Tukey's multiple comparisons test was employed for the post hoc test. Pearson correlation analysis was used for correlation analysis, and the receiver operating characteristic (ROC) curve was adopted for diagnostic analysis. Logistic regression analysis was adopted to derive combined diagnostic factors to conduct ROC combined diagnosis. A value of *P* < 0.05 was regarded as statistically significant.

## 3. Results

### 3.1. Comparison of Clinical Baseline Data between Obese and Nonobese PCOS Patients and Healthy Subjects

Firstly, a total of 102 PCOS patients and 100 healthy controls were enrolled as study subjects. We further divided the PCOS patients into the PCOS-obese group (*N* = 42) and the PCOS-nonobese group (*N* = 60) according to the Asia-Pacific criteria (BMI ≥25 indicative of obesity) proposed by WHO 2000 International Obesity Task Force. Simultaneously, the healthy controls were assigned to the control-obese group (*N* = 32) and control-nonobese group (*N* = 68). Analysis and comparison of clinical baseline data revealed that relative to control-obese subjects, obese PCOS patients presented with evident differences in BMI, Ferriman–Gallwey score, HOMA-IR, FINS, T, FSH, LH/FSH, TNF-*α*, and IL-6 levels (all *P* < 0.05, Table 2). Meanwhile, there were no statistical differences in regard to age, TC, TG, FBG, E2, PRL, and LH levels between the two groups (all *P* < 0.05). Similarly, the BMI, the Ferriman–Gallwey score, HOMA-IR, FINS, T, FSH, LH/FSH, TNF-*α*, and IL-6 levels in the PCOS-nonobese group were remarkably different from those in the control-nonobese group (all *P* < 0.05), while there were no significant differences in regard to age, TC, TG, FBG, E2, PRL, and LH (all *P* < 0.05). Furthermore, analyses of clinical baseline data demonstrated obvious differences in BMI, Ferriman–Gallwey score, FINS, T, LH/FSH, TNF-*α*, and IL-6 levels among the PCOS-obese group and PCOS-nonobese group (all *P* < 0.05).

### 3.2. miR-124-3p Combined with ANGPTL2 Assists the Diagnosis of Obese and Nonobese PCOS

**Figure 1:**
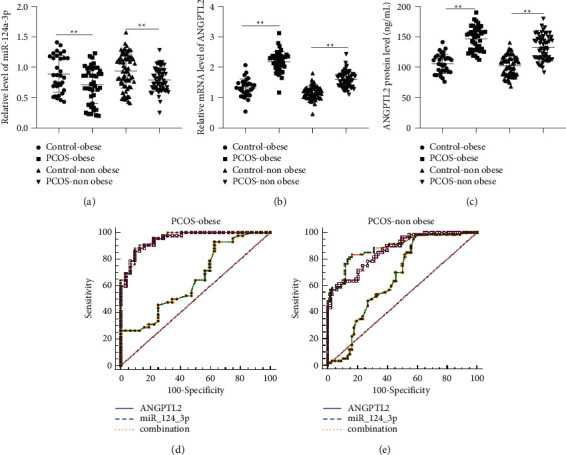
Expression patterns of miR-124-3p and ANGPTL2 in the serum of obese and nonobese PCOS patients and their clinical diagnostic efficacy. (a, b) Expression patterns of miR-124-3p and ANGPTL2 in serum of obese and nonobese PCOS patients (*N* = 102) and healthy subjects (control, *N* = 100) were detected by RT-qPCR. (c) Expression patterns of ANGPTL2 in the serum of obese/nonobese PCOS patients (*N* = 42/60) and healthy obese/nonobese subjects (*N* = 32/68) were detected by ELISA. (d, e) ROC curve was adopted to analyze serum miR-124-3p or/and ANGPTL2 in the diagnosis of obese and nonobese PCOS. Data among multiple groups were analyzed by one-way ANOVA, followed by Tukey's multiple comparisons test. ^*∗∗*^*P* < 0.01.

Expression patterns of miR-124-3p and ANGPTL2 in obese and nonobese PCOS patients and healthy controls were detected by RT-qPCR. It was found that miR-124-3p expression levels were significantly downregulated in the serum of PCOS-obese patients compared to those in the control-obese group; serum miR-124-3p levels were further decreased, and ANGPTL2 levels were elevated in the PCOS-nonobese group compared with those in the control-nonobese group; serum miR-124-3p levels were lower and ANGPTL2 levels were higher in the PCOS-obese group relative to those in the PCOS-nonobese group (Figures 1(a)–1(c), *P* < 0.01). Additional analyses of the ROC curve of miR-124-3p or/and ANGPTL2 revealed the area under the curve (AUC) of miR-124-3p in serum for the diagnosis of obese PCOS was 0.6481, the cutoff value was 1.100, the specificity was 75.00%, and the sensitivity was 92.86%. Meanwhile, the AUC of ANGPTL2 in serum for the diagnosis of obese PCOS was 0.951, the cutoff value was 125.1, the specificity was 90.63%, and the sensitivity was 85.71%. The AUC of the combination of miR-124-3p and ANGPTL2 in serum for the diagnosis of obese PCOS was 0.955, the specificity was 90.63%, and the sensitivity was 88.10% (Figure 1(d)). Furthermore, the AUC of miR-124-3p in serum for predicting nonobese PCOS was 0.6612, the cutoff value was 1.085, the specificity was 86.76%, and the sensitivity was 98.33%. The AUC of ANGPTL2 in serum for predicting nonobese PCOS patients was 0.866, the cutoff value was 122.3, the specificity was 92.65%, and the sensitivity was 61.67%. Lastly, the AUC of the combination of miR-124-3p and ANGPTL2 for predicting nonobese PCOS patients was 0.889, the specificity was 83.82%, and the sensitivity was 83.33% (Figure 1(e)). These findings indicated that miR-124-3p and ANGPTL2 were significantly abnormally expressed in the serum of PCOS patients, and the combination of miR-124-3p and ANGPTL2 had higher AUC, specificity, and sensitivity than the individual diagnostic value of miR-124-3p or ANGPTL2 for obese/nonobese PCOS.

### 3.3. Correlation between the Expression of miR-124-3p and ANGPTL2 with Clinicopathological Features of Obese and Nonobese PCOS

Thereafter, we analyzed the correlation between the expression of miR-124-3p and ANGPTL2 in serum with the clinicopathological characteristics of obese and nonobese PCOS patients, including general condition, glucose metabolism index, lipid metabolism index, and sex hormone levels. In obese PCOS patients, the expression of miR-124-3p was inversely linked with the Ferriman–Gallwey score, FINS, and serum T (*P* < 0.05), while being positively correlated with PRL (*P* < 0.05), but not with other indexes; in nonobese PCOS patients, miR-124-3p was negatively related to the Ferriman–Gallwey score and serum T (*P* < 0.05) and positively correlated with PRL (*P* < 0.05), but not with other parameters (Table 3). Meanwhile, in obese PCOS patients, serum ANGPTL2 levels were markedly positively correlated with FINS and serum T (all *P* < 0.05), and negatively correlated with PRL (*P* < 0.05), but not with other indexes; in nonobese PCOS patients, ANGPTL2 was positively associated with the Ferriman–Gallwey score and FINS (all *P* < 0.05), inversely correlated with PRL (all *P* < 0.05), but not with other parameters (Table 4).

### 3.4. Correlation between miR-124-3p/Angptl2 and Inflammatory Factors in Obese and Nonobese PCOS

**Figure 2:**
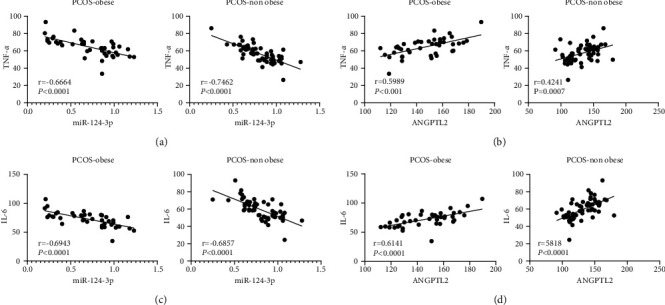
Correlation between serum miR-124-3p and ANGPTL2 with inflammatory factors in PCOS patients. (a–d) Correlation between serum miR-124-3p or ANGPTL2 level and TNF-*α* or IL-6 in PCOS patients. The correlation of data was carried out using Pearson correlation analysis.

Existing evidence indicates that the elevation of TNF-*α* and IL-6 levels is directly associated with the occurrence and development of PCOS [30]. Accordingly, the levels of TNF-*α* and IL-6 in the serum of obese and nonobese PCOS patients were detected using ELISA, followed by analyses of their correlations with serum miR-124-3p and ANGPTL2 mRNA levels. In obese and nonobese PCOS patients, miR-124-3p was negatively correlated with TNF-*α* and IL-6 (Figures 2(a), 2(c), all *P* < 0.01), whereas ANGPTL2 levels were positively correlated with TNF-*α* and IL-6 (Figures 2(b), 2(d), all *P* < 0.01).

### 3.5. miR-124-3p Targeted ANGPTL2

**Figure 3:**
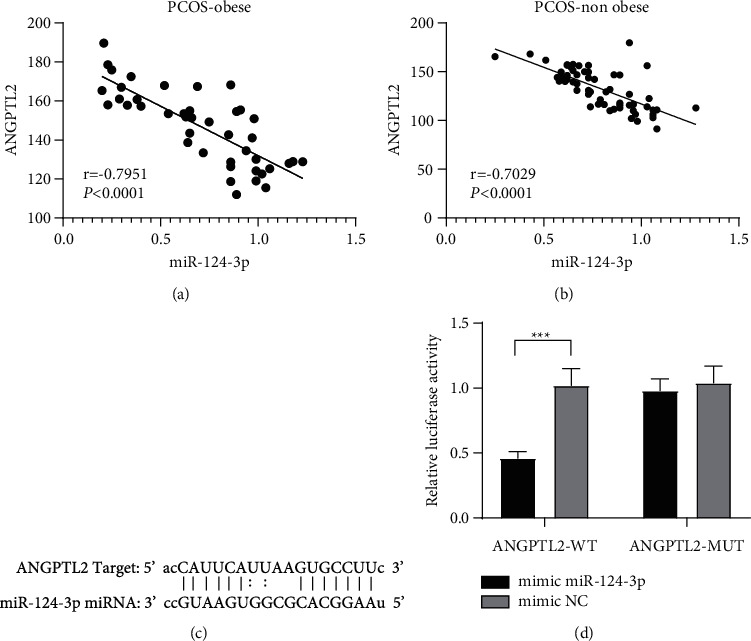
miR-124-3p targeted ANGPTL2. (a, b) The relationship between miR-124-3p and ANGPTL2 mRNA level in obese and nonobese PCOS patients. (c) The binding sites between miR-124-3p and ANGPTL2 predicted using the StarBase database. (d) The binding relationship of miR-124-3p and ANGPTL2 was detected with a dual-luciferase assay. Pearson correlation analysis was used in panels A/B, and the nonpaired *t*-test was used in panel D. ^*∗∗∗*^*P* < 0.001.

The abovementioned findings indicated that miR-124-3p was poorly expressed in PCOS patients, and ANGPTL2 was highly expressed in PCOS patients. Therefore, we analyzed the correlation between the expression patterns of miR-124-3p and ANGPTL2 in obese and nonobese PCOS patients and found that miR-124-3p was significantly negatively correlated with ANGPTL2 (*r* = −0.7951/−0.7029, all *P* < 0.001) (Figures 3(a)-3(b)), which suggested that miR-124-3p might interact with ANGPTL2. Therefore, the binding sites between ANGPTL2 and miR-124-3p were predicted using the StarBase database (Figure 3(c)), suggesting that miR-124-3p and ANGPTL2 might have a targeted binding relationship. Subsequently, the targeted relationship between miR-124-3p and ANGPTL2 was verified by a dual-luciferase assay. Compared with the PGL-ANGPTL2-WT and mimic NC cotransfection group, the fluorescence enzyme activity of KGN cells of the PGL-ANGPTL2-WT and miR-124-3p mimic cotransfected group was significantly decreased (*P* < 0.01) (Figure 3(d)). Together, these findings suggested that ANGPTL2 might play a regulatory role in PCOS as a target gene of miR-124-3p.

## 4. Discussion

As the most prevalent endocrinopathy in women of reproductive age, PCOS exerts a heavy burden on society with prevalence reaching up to 10% [31]. Further adding to the plight of the patients, PCOS accounts for approximately 75% of clinically diagnosed anovulatory infertility [32]. Meanwhile, the hard-done work of our peers has shown that ANGPTL2 and miR-124-3p play an essential role in PCOS [23,27]. Accordingly, the current study set out to explore the clinical roles of serum miR-124-3p/ANGPTL2 in PCOS, and our findings revealed that high expression of ANGPTL2 and low expression of miR-124-3p could assist the diagnosis of obese and nonobese PCOS.

PCOS is a well-characterized endocrine disorder in reproductive-aged women that leads to hormonal, metabolic, and reproductive abnormalities [33], and accompanied by comorbidities such as dyslipidemia, obesity, and insulin resistance, and clinical manifestations including menstrual abnormalities, acne, and hirsutism [4]. The latter is defined as the presence of terminal hair with male distribution in women and is particularly evaluated using the Ferriman–Gallwey scoring system [34]. Herein, our findings demonstrated that compared with healthy subjects, obese and non–obese PCOS patients presented with higher BMI, increased Ferriman–Gallwey score, in addition to elevated HOMA-IR, FINS, total T, LH/FSH, and TNF-*α* and IL-6 levels, and lower FSH levels. This may be attributed to the dysfunction of insulin receptor signal transduction in PCOS patients. As insulin resistance is an important pathogenesis of PCOS, compensatory hyperinsulinemia precipitates the excessive proliferation of follicle membrane cells and stromal cells, augmenting the synthesis and secretion of androgens, such that these high levels of androgens further reduce insulin sensitivity and aggravate insulin resistance in the body [35]. On the other hand, insulin sensitizers, such as myo-inositol (MI) and D-chiroinositol (DCI), can significantly alleviate insulin resistance in PCOS, thereby ameliorating PCOS [36]. Additionally, these two inositol isoforms are highly effective in improving ovarian function and metabolism in PCOS patients [37]. E_2_ is a natural estrogen secreted by mature ovarian follicles and is normally associated with the development of the female reproduction system and menstrual cycle regulation. However, estrogen levels are often fluctuating without cycle in PCOS patients. Meanwhile, testosterone (T) is established as the chief source of androgen in PCOS patients, which is primarily secreted by the adrenal gland and ovary. Therefore, T was regarded as the main biochemical index for the diagnosis of PCOS in our study. However, long-term clinical data indicate that E_2_ and T are not sensitive to the diagnosis of PCOS [38]. Another biochemical indexes in our study, FSH and LH, are glycoprotein hormones that act directly on the ovary and improve the activity of granulosa cells and accelerate follicular development. Interestingly, there is evidence to suggest that primary hypothalamic insufficiency serves as the pathogenesis of PCOS. Moreover, a large proportion of PCOS patients present with abnormal hypothalamic pituitary ovarian axis function, which promotes the continuous increase in serum LH levels. Together, excessive LH and insulin stimulate ovarian theca cells and stromal cells in tandem, resulting in excessive follicle recruitment, inhibition of FSH synthesis and secretion, follicular development stagnation, and selection disorder, consequently contributing to anovulation and polycystic ovary formation [38, 39]. Furthermore, PCOS patients are known to exhibit increased levels of TNF-*α* and IL-6 relative to controls, which is indicative of a potential state of chronic subclinical inflammation. Accordingly, it would be plausible to suggest that improving the levels of inflammatory factors in PCOS patients may function as a potential therapeutic target.

Normally, follicular development not only is dependent on hormonal regulation and signal transduction but also requires vasculature supply to maintain adequate nutrition. Interestingly, the focus of our study, ANGPTL1 is closely associated with angiogenesis [40–42]. However, there is a lack of knowledge in regard to the role of ANGPTL1 in follicular development. Nevertheless, ANGPTL2 is known to mediate chronic inflammation and promote obesity-associated insulin resistance in adipose tissues [43]. In addition, ANGPTL2 represents a key inflammatory mediator from adipose tissues, and alternation in circulating ANGPTL2 protein levels serves as a marker for metabolic abnormalities, inducing obesity and promoting chronic adipose tissue inflammation and obesity-related systemic insulin resistance [44]. Furthermore, a plethora of studies have documented the upregulation of ANGPTL2 in PCOS [9, 20, 44], which is in accordance with our findings.

What's more, the ANGPTL2 family in conjunction with various miRNAs is implicated in the pathogenesis of PCOS [45, 46]. However, the expressions and the diagnostic values of ANGPTL2 and miR-124-3p in PCOS remain unclear. Enhancing our understanding of the same, our findings revealed that miR-124-3p was poorly expressed and ANGPTL2 was highly expressed in the serum of obese and nonobese PCOS patients. Consistently, many prior studies have come across significant downregulation of miR-124-3p and marked upregulation of ANGPTL2 in PCOS patients [23, 27]. Together, these findings and evidence indicate that miR-124-3p and ANGPTL2 were abnormally expressed in obese and nonobese PCOS patients.

miRNAs expressed in body fluids, including blood, have been previously adopted as reliable biomarkers and predictors of treatment response in a wide array of diseases, such as cancers, heart disease, and immune-related diseases [47]. Unsurprisingly, numerous miRNAs are abnormally expressed in PCOS ovaries and implicated in intra-ovarian inflammation and insulin sensitivity [48, 49]. The study carried out by Sørensen et al. pointed out that miR-24-3p, miR-29a, miR-151-3p, and miR-574-3p levels were all diminished in PCOS patients, and miR-518f-3p was differentially expressed, whereas miR-518f-3p alone or in combination with these four miRNAs could diagnose PCOS, and miRNAs in specific follicular fluid were associated with phenotypic characteristics of PCOS and insulin resistance [50]. Meanwhile, there is much evidence to suggest that miR-146a, miR-370, miR-155, and miR-708 are linked with insulin resistance [51]. In addition, miR-122, miR-193b, and miR-194 are known to be elevated in the serum of PCOS patients and further exhibit modest correlations with HOMA-IR [52]. On a separate note, obesity not only enhances insulin resistance but also serves as a pathogenic factor, resulting in endocrine disorders due to altered hormonal metabolism in adipocytes. Furthermore, adipose tissue contributes to sustaining a low level of systemic inflammation, which further underscores the significance of obesity in PCOS [53, 54]. As indicated above, PCOS is regarded as a chronic inflammatory state, accompanied by elevated white blood cells, pro-inflammatory cytokines, and white blood cell counts [13–15]. What's noteworthy is that miRNAs possess the ability to downregulate the expression of inflammatory genes in vitro and in vivo by targeting inflammatory mediators to regulate inflammatory responses [55]. Similarly, there is evidence to suggest that miR-124-3p is associated with inflammation [16, 56–58], but studies about PCOS are scarce. In our efforts, we explored the expression of miR-124-3p in the serum of PCOS patients and its clinical diagnostic value and uncovered that serum miR-124-3p was downregulated in PCOS patients, which was consistent with preceding findings [27]. Additionally, our findings indicated that serum miR-124-3p level <1.075 could assist the PCOS diagnosis. ANGPTL2 is a kind of secretory glycoprotein, which has a certain homology and similar domain with angiopoietin but has a wider diversity in function. Besides, ANGPTL2 has been reported in a series of metabolic disorders such as obesity, inflammation, insulin resistance, lipid metabolism disorder, and malignancies [21, 59–61]. However, it remains unknown whether ANGPTL2 can be adopted as a new molecular target to treat PCOS by reducing its expression or inhibiting its activity. As elaborated earlier, elevated TNF-*α* and IL-6 levels are directly associated with PCOS occurrence and development [30]. Therefore, we analyzed the correlation between miR-124-3p and ANGPTL2 expressions in serum and TNF-*α* and IL-6 levels in serum of obese and nonobese PCOS patients and unraveled that miR-124-3p was negatively correlated with TNF-*α* and IL-6, while ANGPTL2 was positively correlated with TNF-*α* and IL-6. Moreover, our findings illustrated the presence of a binding relationship between miR-124-3p and ANGPTL2. Together, these data indicate that ANGPTL2 might play a regulatory role in PCOS as a target gene of miR-124-3p.

In summary, our study was the first-of-its-kind to highlight the negative correlation between serum miR-124-3p and ANGPTL2 in obese and nonobese PCOS patients and that miR-124-3p combined with ANGPTL2 could assist the diagnosis of PCOS. However, we only preliminarily explored the mechanism of ANGPTL2 and miR-124-3p in PCOS by predicting the binding sites between the two using the StarBase database and verifying the targeted relationship in KGN cells, but the specific molecular mechanism by which miR-124-3p affects the onset and development of PCOS through ANGPTL2 remains unclear and requires further investigation.

## Figures and Tables

**Table 1:** Primer sequences.

Gene	Forward 5′-3′	Reverse 5′-3′
miR-124-3p	TCTTTAAGGCACGCGGTG	TATGGTTTTGACGACTGTGTGAT
ANGPTL2	GAACCGAGTGCATAAGCAGGA	GTGACCCGCGAGTTCATGTT
GAPDH	GGAGCGAGATCCCTCCAAAAT	GGCTGTTGTCATACTTCTCATGG
U6	CTCGCTTCGGCAGCACA	AACGCTTCACGAATTTGCGT

**Table 2:** Comparison of clinical baseline data between obese and nonobese PCOS patients and healthy subjects.

Feature	Control-obese	PCOS-obese	Control-nonobese	PCOS-nonobese	*Pa*	*Pb*	*Pc*
	(*N* = 32)	(*N* = 42)	(*N* = 68)	(*N* = 60)			
General comparison							
Age (year)	26 ± 3.6	26 ± 3.8	26 ± 3.5	26 ± 3.7	>0.9999	>0.9999	>0.9999
BMI (kg/m^2^)	26.46 ± 0.94	30.02 ± 1.66	21.03 ± 1.45	23.07 ± 1.40	<0.0001	<0.0001	<0.0001
Ferriman–Gallwey score	4.87 ± 1.74	7.79 ± 2.52	4.13 ± 1.67	5.92 ± 1.29	<0.0001	<0.0001	<0.0001
Lipid metabolism index							
TC (mmol/L)	4.52 ± 0.86	4.86 ± 0.88	4.48 ± 0.63	4.63 ± 0.72	0.2198	0.6735	0.4269
TG (mmol/L)	1.28 ± 0.24	1.33 ± 0.28	1.22 ± 0.21	1.29 ± 0.25	0.8159	0.3643	0.845
Glucose metabolism index							
HOMA-IR	2.47 ± 0.39	2.87 ± 0.66	2.39 ± 0.35	2.83 ± 0.65	0.0083	<0.0001	0.982
FBG (mmol/L)	5.13 ± 0.82	5.52 ± 0.94	4.82 ± 0.78	5.09 ± 0.84	0.2031	0.2734	0.058
FINS (mU/L)	10.86 ± 1.84	12.48 ± 2.03	9.96 ± 1.64	11.36 ± 2.01	0.0016	0.0002	0.0171
Sex hormone levels							
T (ng/mL)	0.46 ± 0.12	0.63 ± 0.24	0.41 ± 0.12	0.53 ± 0.16	<0.0001	0.0003	0.0141
E_2_ (pg/mL)	48.34 ± 8.62	52.32 ± 9.03	48.26 ± 8.26	50.26 ± 8.86	0.2074	0.5616	0.6387
PRL (ng/mL)	11.86 ± 2.42	11.16 ± 1.16	11.68 ± 1.26	11.38 ± 1.08	0.1672	0.6438	0.8731
LH (mIU/mL)	6.46 ± 1.62	7.62 ± 2.61	5.84 ± 1.58	6.68 ± 2.48	0.0946	0.1172	0.126
FSH (mIU/mL)	6.35 ± 1.28	5.52 ± 1.12	6.64 ± 1.29	5.59 ± 1.12	0.0194	0.0067	0.31
LH/FSH	1.12 ± 0.38	1.28 ± 0.31	0.88 ± 0.13	1.08 ± 0.23	<0.0001	<0.0001	<0.0001
Inflammatory factor levels							
TNF-*α* (pg/mL)	49.36 ± 4.26	64.68 ± 10.42	46.32 ± 4.03	57.28 ± 9.64	<0.0001	<0.0001	<0.0001
IL-6 (pg/mL)	45.62 ± 5.86	72.36 ± 12.64	38.69 ± 5.26	59.84 ± 11.08	<0.0001	<0.0001	<0.0001

*Note.* Pa, PCOS-obese group versus control-obese group; Pb, PCOS-nonobese group versus control-nonobese group; Pc, PCOS-obese group versus PCOS-nonobese group. *P* < 0.05 indicated statistical significance. BMI = body mass index; TC = total cholesterol; TG = total triglyceride; HOMA-IR = HOMA insulin resistance; FBG = fasting blood glucose; FINS = fasting insulin; T = total testosterone; E2 = estradiol; PRL = prolactin; LH = luteinizing hormone; FSH = follicle-stimulating hormone.

**Table 3:** Relationship between the expression of miR-124-3p in serum and the clinicopathological features of obese and nonobese PCOS patients.

Parameters	PCOS-obese (*N* = 42)		PCOS-nonobese (*N* = 60)	
	Pearson *r*	*P*	Pearson *r*	*P*
General comparison				
Age	−0.084	0.597	−0.0292	0.8248
BMI	0.0525	0.7411	0.0861	0.513
Ferriman–Gallwey score	−0.3935	0.0099	−0.4104	0.0011
Lipid metabolism index				
TC	−0.0758	0.6333	−0.0312	0.8129
TG	−0.0768	0.6288	−0.0308	0.8155
Glucose metabolism index				
HOMA-IR	−0.267	0.0874	−0.148	0.259
FBG	−0.0769	0.6285	0.0322	0.8072
FINS	−0.3633	0.018	−0.2541	0.0501
Sex hormone levels				
T	−0.4586	0.0023	−0.3685	0.0038
E_2_	−0.0753	0.6354	−0.03	0.8202
PRL	0.6254	<0.0001	0.6664	<0.0001
LH	−0.0755	0.6346	−0.0299	0.8204
FSH	0.2612	0.0947	0.0421	0.7492
LH/FSH	−0.2115	0.1787	0.0045	0.9729

*Note.* Pearson correlation coefficient was used to analyze the data. BMI = body mass index; TC = total cholesterol; TG = total triglyceride; HOMA-IR = HOMA insulin resistance; FBG = fasting blood glucose; FINS = fasting insulin; T = testosterone; E2 = estradiol; PRL = prolactin; LH = luteinizing hormone; FSH = follicle-stimulating hormone.

**Table 4:** Relationship between the expression of ANGPTL2 in serum and the clinicopathological features of obese and nonobese PCOS patients.

Parameters	PCOS-obese (*N* = 42)		PCOS-nonobese (*N* = 60)	
	Pearson *r*	*P*	Pearson *r*	*P*
General comparison				
Age	0.0841	0.5966	0.0002	0.9985
BMI	0.0064	0.968	0.045	0.7333
Ferriman–Gallwey score	0.2012	0.2014	0.2853	0.0271
Lipid metabolism index				
TC	0.0787	0.6205	−0.0101	0.939
TG	0.0792	0.6181	−0.0094	0.9429
Glucose metabolism index				
HOMA-IR	0.2072	0.1879	0.0282	0.8307
FBG	0.0795	0.6168	−0.0728	0.5804
FINS	0.38	0.013	0.2734	0.0345
Sex hormone levels				
T	0.4227	0.0053	0.2186	0.0934
E_2_	0.0784	0.6218	−0.0108	0.935
PRL	−0.3808	0.0129	−0.4387	0.0005
FSH	−0.3625	0.0183	−0.161	0.219
LH	0.0785	0.6212	−0.011	0.9335
LH/FSH	0.2569	0.1005	0.0171	0.8967

*Note.* Pearson correlation coefficient was used to analyze the data. BMI = body mass index; TC = total cholesterol; TG = total triglyceride; HOMA-IR = HOMA insulin resistance; FBG = fasting blood glucose; FINS = fasting insulin; T = total testosterone; E2 = estradiol; PRL = prolactin; LH = luteinizing hormone; FSH = follicle-stimulating hormone.

## Data Availability

The data that support the findings of this study are available from the corresponding author upon reasonable request.
